# Lawson Tait on Bacterial Pathology

**Published:** 1894-12

**Authors:** 


### Lawson Tait on Bacterial Pathology.

Mr. Lawson Tait is probably the most aggressive opponent of the germ theory of inflammation. He says that during his professional life he has learned and unlearned some four or five theories of inflammation, and predicts that the present prevalent theory—a phase of lunacy; coccophobia, he calls it—will soon go the way of the other theories.—*Medical Age.*

